# Tumor immune microenvironment in therapy‐naive esophageal adenocarcinoma could predict the nodal status

**DOI:** 10.1002/cam4.5386

**Published:** 2022-10-25

**Authors:** Andromachi Kotsafti, Matteo Fassan, Francesco Cavallin, Valentina Angerilli, Luca Saadeh, Matteo Cagol, Rita Alfieri, Pierluigi Pilati, Carlo Castoro, Ignazio Castagliuolo, Melania Scarpa, Marco Scarpa

**Affiliations:** ^1^ Laboratory of Advanced Translational Research Veneto Institute of Oncology, IOV – IRCCS Padua Italy; ^2^ Department of Medicine DIMED University of Padua Padua Italy; ^3^ Oncological Surgery Unit Veneto Institute of Oncology, IOV – IRCCS Padua Italy; ^4^ Independent Statistician Solagna Italy; ^5^ Chirurgia Generale 3 University Hospital of Padua Padua Italy; ^6^ Department of Upper GI Surgery Humanitas Research Hospital‐Humanitas University Rozzano Italy; ^7^ Department of Molecular Medicine University of Padua Padua Italy

**Keywords:** biomarkers, esophageal adenocarcinoma, immune surveillance, nodal metastasis, tumor immune microenvironment

## Abstract

**Background:**

Currently, preoperative staging of esophageal adenocarcinoma (EAC) has modest reliability and accuracy for pT and pN stages prediction, which heavily affects overall survival. The interplay among immune checkpoints, oncogenes, and intratumoral and peritumoral immune infiltrating cells could be used to predict loco‐regional metastatic disease in early EAC.

**Methods:**

We prospectively evaluated immune markers expression and oncogenes status as well as intratumoral and peritumoral immune infiltrating cells populations in esophageal mucosa samples obtained from neoadjuvant therapy‐naïve patients who had esophagectomy for EAC.

**Results:**

Vascular invasion and high infiltration of lamina propria mononuclear cells resulted associated with nodal metastasis. Low infiltration of activated CD8^+^CD28^+^ T cells was observed in both intratumoral and peritumoral mucosa of patients with nodal metastasis. Low levels of CD69, MYD88, and TLR4 transcripts were detected in the intratumoral specimen of patients with lymph node involvement. Receiver operating characteristic curve analysis showed good accuracy for detecting nodal metastasis for all the markers tested. Significant lower infiltration of CD8 T cells and M1 macrophages and a lower expression of CD8A, CD8B, and TBX21 were found also in Esophageal Adenocarcinoma TCGA panCancer Atlas in the normal tissue of patients with nodal metastasis.

**Conclusions:**

Our data suggest that immune surveillance failure is the main driver of nodal metastasis onset. Moreover, nodal metastasis containment also involves the immune microenvironment of the peritumoral healthy tissue.

## Graphical abstract

Low infiltration of activated CD8^+^CD28^+^ T cells was observed in both intratumoral and peritumoral mucosa of patients with nodal metastasis suggesting that immune surveillance failure is the main driver of nodal metastasis onset.

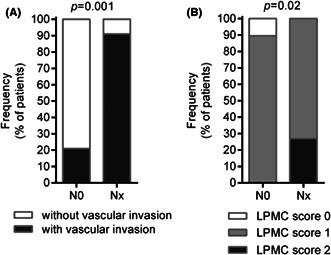

## 1 INTRODUCTION

Esophageal adenocarcinoma (EAC) is an increasingly frequent malignancy characterized by a poor prognosis. Multimodality therapy protocols combining neoadjuvant radiation and/or chemotherapy followed by surgery are the present treatment option.^1^, ^2^ Neoadjuvant chemoradiotherapy successfully down‐staged locally advanced EAC patients,^3^, ^4^, ^5^, ^6^ but according to the NCCN guidelines patients with early EAC (pT1N0 or pT2N0) usually do not have neoadjuvant therapy.^7^

The accuracy of the preoperative evaluations of several staging parameters is often inadequate with several underestimations.^8^ Endoscopic ultrasound scan shows poor accuracy in preoperative staging of the esophagogastric junction and esophageal adenocarcinoma. In fact, for node (N) staging, sensitivity is 77.3% and specificity 67.4%, with an accuracy of 77.9%.^9^ Baseline SUV_max_ of 18‐fluorine‐fluorodeoxyglucose positron emission tomography/computed tomography (^18^F‐FDG PET/CT) exhibits a high predictive value of the preoperative CT stage, as it can predict a locally advanced tumor with high accuracy.^10^ Preoperative staging of esophageal adenocarcinoma has modest reliability and accuracy for pT and pN stages prediction, with as much as 25% of patients having conflicting clinical and pathological staging, which heavily affects overall survival.^11^

The immune system can recognize and eliminate tumor cells based on their expression of tumor‐specific antigens.^12^ The antitumor action of the immune system is exerted by the activation of T lymphocytes that infiltrate the neoplasm, inhibiting its proliferation. The tumor cell can be recognized and activate the lymphocytes through a two‐signal model that involves HLA molecules and costimulatory molecules. On the other hand, immune checkpoints such as programmed death PD‐1 have the main function of inhibiting T cell activity^13^, ^14^, ^15^, ^16^ through interaction with its ligands PD‐L1 and PD‐L2.^17^

Several oncogenes may modulate the tumor microenvironment. Mutations in *TP53* are a negative predictor of survival after surgery^18^ and CD80 expression shows a robust correlation with *TP53* activation confirming the connection between TP53 and immune surveillance in cancer.^19^ The overexpression of HER2 is found in about 20% of gastroesophageal carcinomas and is associated with a poor prognosis.^20^ *BRAF* encodes a cytoplasmic serine/threonine kinase that acts as an oncogene.^21^ Sommerer et al. detected activating *BRAF* mutations in 11% of adenocarcinomas.^22^ Mismatch repair (MMR) genes are involved in the recognition and repair of the nucleotide mismatch during DNA replication^23^ and their alterations result in microsatellite instability (MSI). The prevalence of MSI in EAC ranges from 5% to 10%.^24^

Therefore, we hypothesize that the interplay among immune checkpoints, oncogenes, and intratumoral and peritumoral immune microenvironment could be used to implement cancer staging to choose the best therapy in the early EAC stage.

## 2 METHODS

### 2.1 Study design

A prospective cohort of 206 patients who had esophagectomy for EAC was examined in the MICCE1 project and among them, all the consecutive 30 patients with therapy‐naive pTis, pT1, pT2, or pT3 EAC were selected. Esophageal samples were obtained from surgical specimens of normal and neoplastic mucosa and processed for subsequent analysis as described below. The diagnosis was supported by histological, clinical, and radiological parameters. The study was performed according to the guidelines of the Declaration of Helsinki, all patients signed the informed consent, and IRB approval (Veneto Institute of Oncology, Padua, Italy) was obtained.

### 2.2 Preoperative staging and neoadjuvant therapy

The staging was performed with upper gastrointestinal endoscopy, thorax, and abdominal CT scan, and ^18^F‐FDG PET/CT. Patients staged below cT3N_0_ or any cT pN1 were considered suitable for surgery alone. Patients staged below cT3N0 were eligible for resection when there was no evidence of distant metastases or locally advanced tumors with evident periesophageal involvement at staging.

### 2.3 Surgery

Surgical techniques details have been described elsewhere.^25^ In brief, the Ivor‐Lewis esophagectomy was performed, through a laparotomy and then a right thoracotomy, for cancers of the esophagogastric junction and mid‐lower esophagus. To avoid neoplastic involvement of the resection margins, at least 6–8 cm of the healthy esophagus was removed above the proximal edge of the tumor. Lymph nodes (LN) were dissected en bloc. Patients were examined at scheduled intervals after 1, 3, 6, and 12 months and every 6–12 months thereafter. Fresh specimens were immediately frozen in liquid nitrogen or fixed in formalin for subsequent analysis.

### 2.4 Histopathology

Histopathology was performed according to international protocols and with a standardized protocol of sampling and processing of the biospecimens. Two gastrointestinal pathologists jointly reassessed all cases. The 7th edition of the TNM classification was used to evaluate the pathological Nodal status (pN0, pN1). For this study, the number of metastatic LN and their site were also examined.^26^

### 2.5 Immunohistochemistry

The Bond Polymer Refine Detection Kit (Leica Biosystems) on BOND‐MAX automated IHC stainer (Leica Biosystems) was used for immunohistochemical stainings. The following primary antibodies were used according to the manufacturer's directions: MLH1 (clone ES05, 1:100; Dako), MSH2 (clone FE11, 1:100; Dako), MSH6 (clone EP49, 1:100; Dako), PMS2 (clone EP51, 1:100; Dako), PD‐L1 (clone 22C3, 1:50; Dako), PD‐L2 (clone 176611, 1:1000; R&D Systems, Inc.), p53 (clone DO7, 1:50; Dako), CD80 (clone 37711, 1:40; R&D Systems, Inc.), CD8 (clone C8/144B, 1:200; Dako), LAMP1 (clone H5G11, 1:50; Santa Cruz Biotechnologies) and HER2 (Hercept test; Dako).

### 2.6 Flow cytometry

Fresh mucosa samples were minced and filtered through a sterile Nylon Filter (BD Falcon). The single cell suspension was incubated with human Fc Receptor binding inhibitor (eBioscience) for 20 min, pelleted, and stained in FACS buffer (phosphate‐buffered saline/2% Flow cytometry staining/0.02% sodium azide) for 30 min at 4°C. The following fluorochrome‐conjugated antibodies were used: anti‐human CD8A PE (clone HIT8a), anti‐human CD28 FITC (clone CD28.2), anti‐human CD80 FITC (clone 2D10.4), anti‐human HLA ABC FITC (clone W6/32) all from eBioscience and anti‐pan Cytokeratin PE (clone C‐11; Abcam). Stained samples were acquired on a FACSCalibur based on CellQuest software (Becton Dickinson). The percentage of cells positive for the molecules of interest was reported.

### 2.7 RNA extraction and qRT‐PCR

RNA was purified from snap‐frozen esophageal mucosa using the SV Total RNA Isolation System (Promega) according to the manufacturer's directions. The iScript cDNA Synthesis Kit (Bio‐Rad) was used for complementary DNA synthesis. The ABI PRISM 7000 Sequence Detection System (Applied Biosystems) was utilized to quantify specific mRNA transcripts with SYBR Green PCR Master Mix. The expression of the *ACTB* housekeeping gene was used to normalize the expression of the target molecules. Sequences of PCR primer pairs were for *CD69* fw 5′ CAAGTTCCTGTCCTGTGTGCT 3′ rv 5′ GCCCACTGATAAGGCAATGAG 3′; *CD80* fw 5′ CTCA CTTCTGTTCAGGTGTTATCCA 3′ rv 5′ TCCTTTTGCCA GTAGATGCGA 3′; *TLR4* fw 5′ TTTCCTGCAATGGA TCAAGGA 3′ rv 5′ TTATCTGAAGGTGTTGCACATTCC 3′; *MYD88* fw 5′ GGATGGTGGTGGTTGTCTCT 3′ rv 5′ AGGATGCTGGGGAACTCTTT 3′; *ACTB* fw 5′ CTG GACTTCGAGCAAGAGATG 3′ rv 5′ AGTTGAAGGTAGT TTCGTGGATG 3′.

### 2.8 MSI and *BRAF* mutational status

DNA was isolated from four consecutive 5 μm thick sections obtained from tumor tissue and matched normal mucosa. DNA was extracted from manually micro‐dissected neoplastic cells by using the Mini Amp kit (Qiagen) following the manufacturer's directions. DNA quality was assessed on the TapeStation 4200 microfluidic platform (Agilent Technologies) using the Genomic ScreenTape device (Agilent Technologies), following the manufacturer's instructions. The extracted DNA was analyzed with the MSI Titano kit (Diatech Pharmacogenetics) following the manufacturer's instructions. Exon 15 *BRAF* status was analyzed by conventional Sanger sequencing.^27^

### 2.9 External series

Publicly accessible Esophageal Adenocarcinoma TCGA panCancer Atlas data were considered for external cohort in‐silico analysis.^28^ Seventy tumor specimens and seven paired adjacent normal esophageal tissues from 70 therapy‐naive EAC patients staged pTis, pT1, pT2, or pT3 (*n* = 70, 22 pN0 vs. 48 pNx) were selected. Tumor staging, LN metastasis, and gene mutational status‐related data were extracted through the Computational Biology Center Portal (cBioportal).^29^, ^30^ Data of gene expression were downloaded through UCSC Xena browser.^31^ Gene expression profiles of our panel of selected immune genes were analyzed, and the association between expression or genomic alteration and nodal metastasis was tested with a non‐parametric Mann–Whitney *U* test. The CIBERSORTx web portal (https://cibersortx.stanford.edu)^32^ was exploited to run the validated 22‐phenotype leukocyte signature (LM22) in absolute mode with 1000 permutations and B‐mode batch correction. RSEM normalized gene expression data were utilized as input data. Samples with accurate CIBERSORTx deconvolution (*p* < 0.05) were used for further immunophenotyping analysis.

### 2.10 Statistical analysis

Data are shown as median with interquartile range (IQR) (continuous variables) or frequency with percentage (categorical data) with a descriptive purpose. In all patients, data were compared using the Kruskal‐Wallis test (continuous variables) or Fisher's test (categorical data). Factors associated with LN involvement were investigated with univariate logistic regression models. Receiver operating characteristic (ROC) analysis was used to assess the accuracy of the potential markers for the prediction of the presence of nodal metastasis. Immune cell rate and immune gene mRNA expression levels were used for ROC curve analysis. All tests were two‐sided and a *p*‐value less than 0.05 was considered statistically significant. Statistical analysis was performed using R 3.3 (R Foundation for Statistical Computing).^33^

## 3 RESULTS

### 3.1 Patient characteristics

**FIGURE 1:**
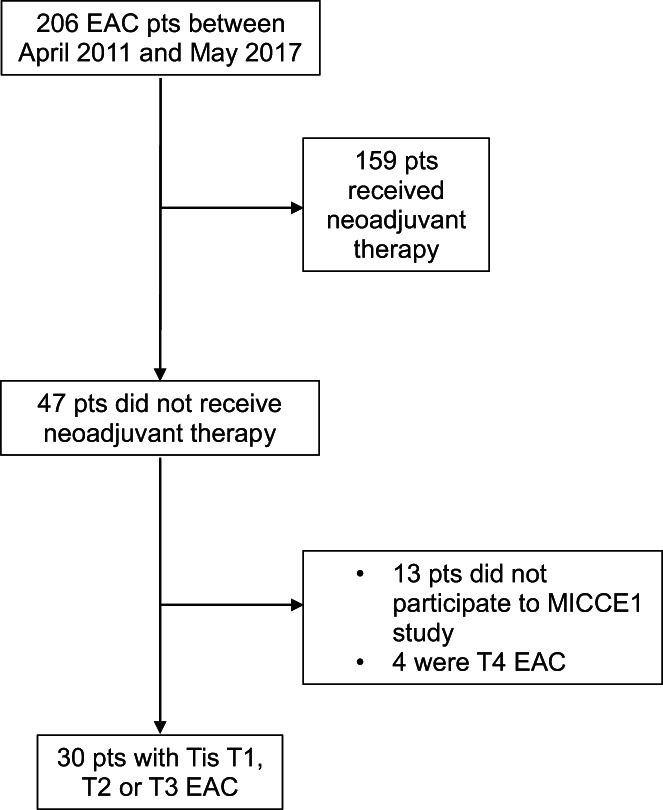
Consort diagram of esophageal adenocarcinoma patients selected for the study. The comparison of the demographic data between the whole group of patients and the selected group was carried out between similarly selected patients.

Between April 2011 and May 2017, 206 consecutive patients with esophageal adenocarcinoma were evaluated for inclusion in the MICCE1 project at the Veneto Institute of Oncology. One hundred fifty‐nine patients treated with neoadjuvant therapy were excluded. In total, a cohort of 30 patients staged pTis, pT1, pT2, or pT3 was included in this study (Figure 1). Patient characteristics are outlined in Table 1. The median age was 71 (IQR 62–78) and 26 of them were males. LN involvement was identified in 11 patients (35%). The comparison of the demographic data between the whole group of patients who had neoadjuvant therapy and accepted to participate to MICCE1 project (*n* = 121) and the selected group only showed an expected difference in terms of T‐category distribution. The group of patients who had neoadjuvant therapy had generally a more advanced ypT stage or, paradoxically, a ypT0 stage after the complete response to neoadjuvant therapy. This comparison is shown in Table S1.

**TABLE 1:** Characteristics of the study population (*n* = 30)

Characteristics	Lymph nodes involvement		*p* values
	Absent (*n* = 19)	Present (*n* = 11)	
Age, median (IQR)	70.5 (IQR 64–78)	76 (IQR 50–80)	0.568
Gender			0.281
Female	4	0	
Male	15	11	
Associated to Barrett epithelium			0.100
Yes	8	1	
No	11	10	
Cancer stage			0.019
Tis	2	0	
T1	9	2	
T2	4	2	
T3	4	7	
Cancer grading			0.701
G1	4	1	
G2	8	7	
G3	7	3	
Nodal metastasis			
Number of resected lymph nodes	23.5 (IQR 18–32.5)	30 (IQR 26.7–36.2)	0.147
Numbers of nodal metastasis	0	3 (IQR 1–7.5)	<0.001
Surgical radicality			0.999
R0	19	11	
R1	0	0	

Abbreviation: IQR, interquartile range.

### 3.2 Intratumoral and peritumoral immune markers as predictors of nodal metastases

**FIGURE 2:**
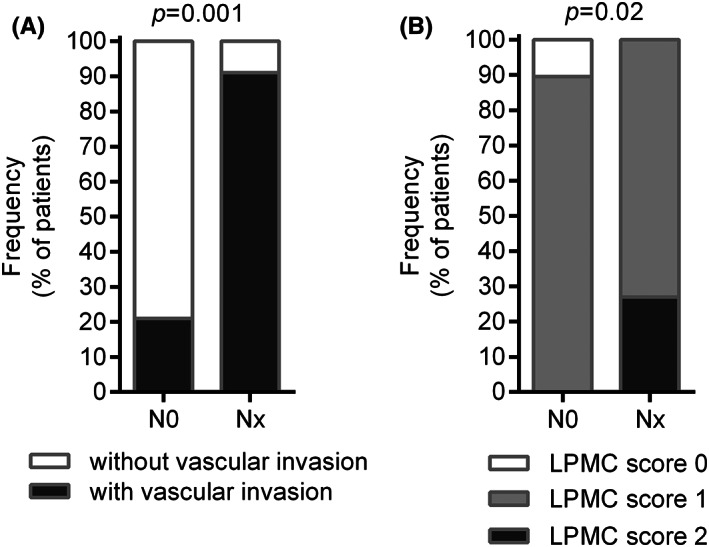
Histochemical analysis of histopathological sections of esophageal adenocarcinoma: (A) Vascular invasion (scored as 0 = absent and 1 = present); (B) infiltrating lamina propria mononuclear cells (scored as 0 = absent, 1 = low, 2 = high).

**FIGURE 3:**
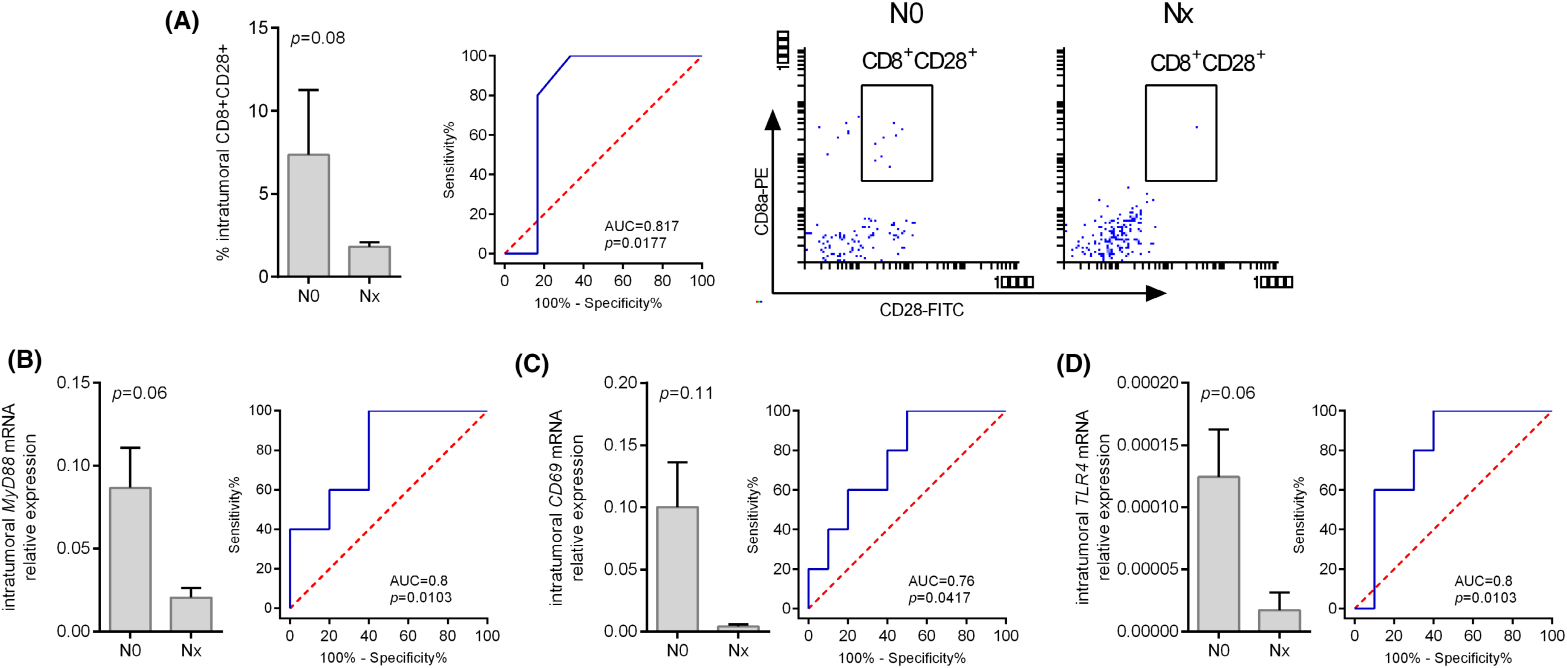
(A) Flow cytometric analysis of CD8^+^CD28^+^ T cells within the tumor. Representative images of flow cytometric analysis of CD8^+^CD28^+^ T cells are shown. The percentage of cells positive for CD8 and CD28 is reported. (B) MYD88 mRNA expression levels within the tumor. (C) TLR4 mRNA expression levels within the tumor. (D) CD69 mRNA expression levels within the tumor. The expression of the ACTB housekeeping gene was used to normalize the expression of the target genes. Data are reported as 2^[CT(housekeeping) − CT(target)]^.

**FIGURE 4:**
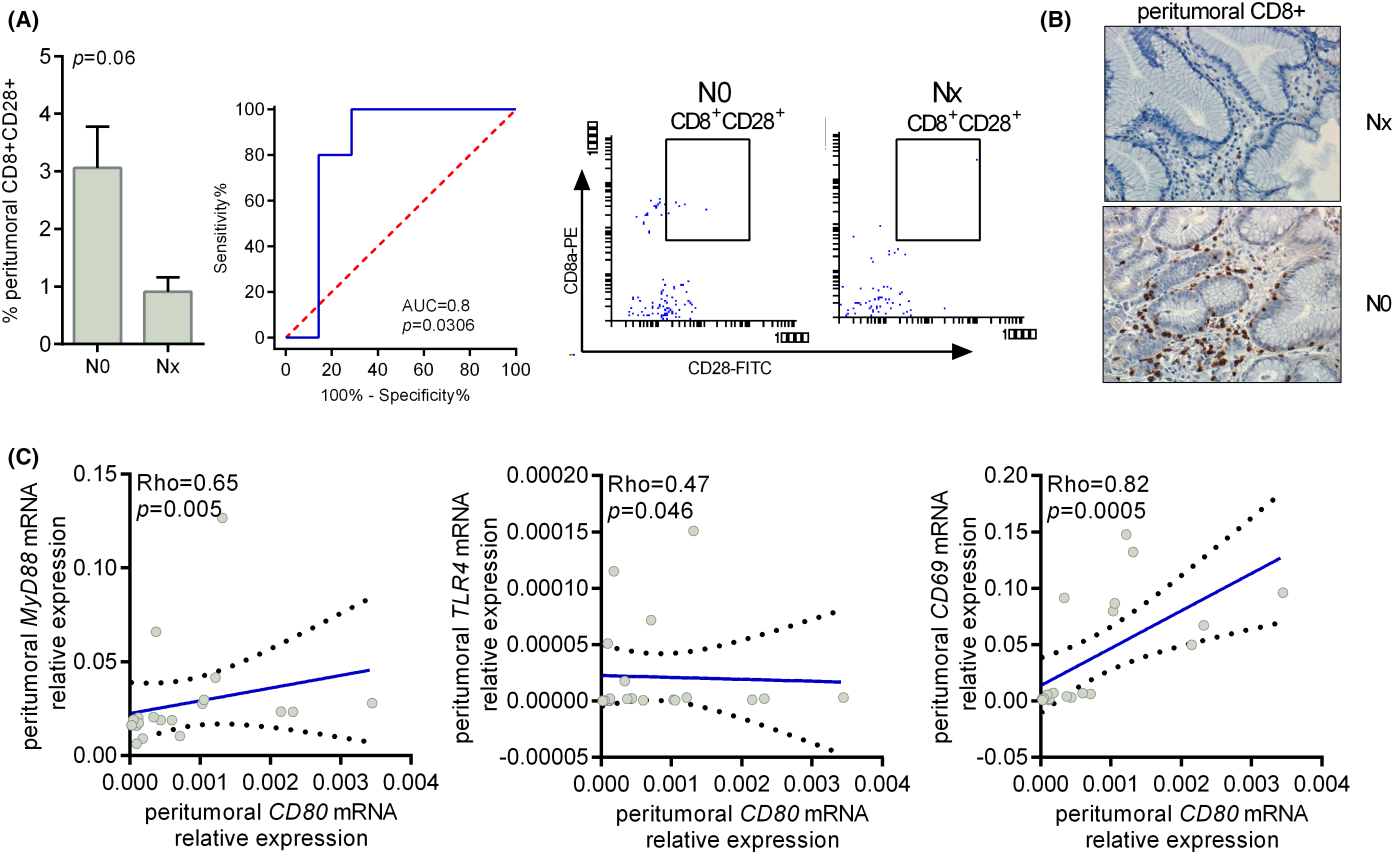
(A) Flow cytometric analysis of CD8^+^CD28^+^ T cells in the peritumoral healthy tissue. The percentage of cells positive for CD8 and CD28 is reported. (B) Representative images of peritumoral CD8^+^ according to nodal status. (C) Correlation between CD80 and MYD88, TLR4, or CD69 mRNA expression levels in the peritumoral healthy tissue. The expression of the ACTB housekeeping gene was used to normalize the expression of the target molecules.

To determine the predictive value of immune markers in the intratumoral and peritumoral esophageal mucosa of therapy‐naïve EAC patients, real‐time qRT‐PCR, flow cytometry as well as histopathology techniques were used to assess the effects of the markers on different LN stages (pN0 and pN1). In our series, no association between a mutation in *TP53*, *BRAF*, MMRd/MSI, or alterations in c‐Myc, p16, and nodal metastasis was observed (data not shown). As shown in Figure 2, LN involvement was associated with vascular invasion (odds ratio [OR] 27.5, 95% confidence interval [CI] 2.6–289; *p* = 0.005) and with high intratumoral infiltration of lymphomononuclear cells (*p* = 0.02). However, nodal metastasis was associated with low levels of activated CD8 T cells expressing CD28 within the tumor (*p* = 0.08). ROC curve showed a good diagnostic accuracy of nodal involvement (AUC [area under the curve] = 0.81 [95% CI: 0.48–0.97], *p* = 0.01) (Figure 3A). Moreover, nodal metastasis tended to be associated with low levels of CD69, MYD88, and TLR4 mRNA expression within the tumor (*p* = 0.11, *p* = 0.06, and *p* = 0.06, respectively). ROC curve analysis of CD69, MYD88 and TLR4 mRNA expression levels showed a good accuracy in diagnosis of nodal involvement (AUC = 0.76 [95% CI: 0.47–0.93], *p* = 0.04; AUC = 0.80 [95% CI: 0.52–0.95], *p* = 0.01; and AUC = 0.80 [95% CI: 0.52–0.95], *p* = 0.01) (Figure 3B–D). Low levels of CD8^+^CD28^+^ T cells were associated with nodal metastasis also when detected in the peritumoral healthy mucosa (*p* = 0.06) (Figure 4A,B). ROC curve analysis activated CD8 T cell infiltration levels and showed good accuracy in the diagnosis of nodal involvement (AUC = 0.80 [95% CI: 0.46–0.97], *p* = 0.03). Besides, in peritumoral healthy mucosa *CD80* mRNA levels directly correlated with *MYD88*, *TLR4*, and *CD69* mRNA levels (Rho = 0.65, *p* = 0.005; Rho = 0.47, *p* = 0.047, and Rho = 0.82, *p* = 0.0005, respectively), indicating a possible pathway leading to CD8 T cells activation (Figure 4C and Table S4). Finally, the small sample size did not allow for evidence of any correlation between immune parameters and cancer recurrence as shown in Figure S1.

### 3.3 External cohort

**FIGURE 5:**
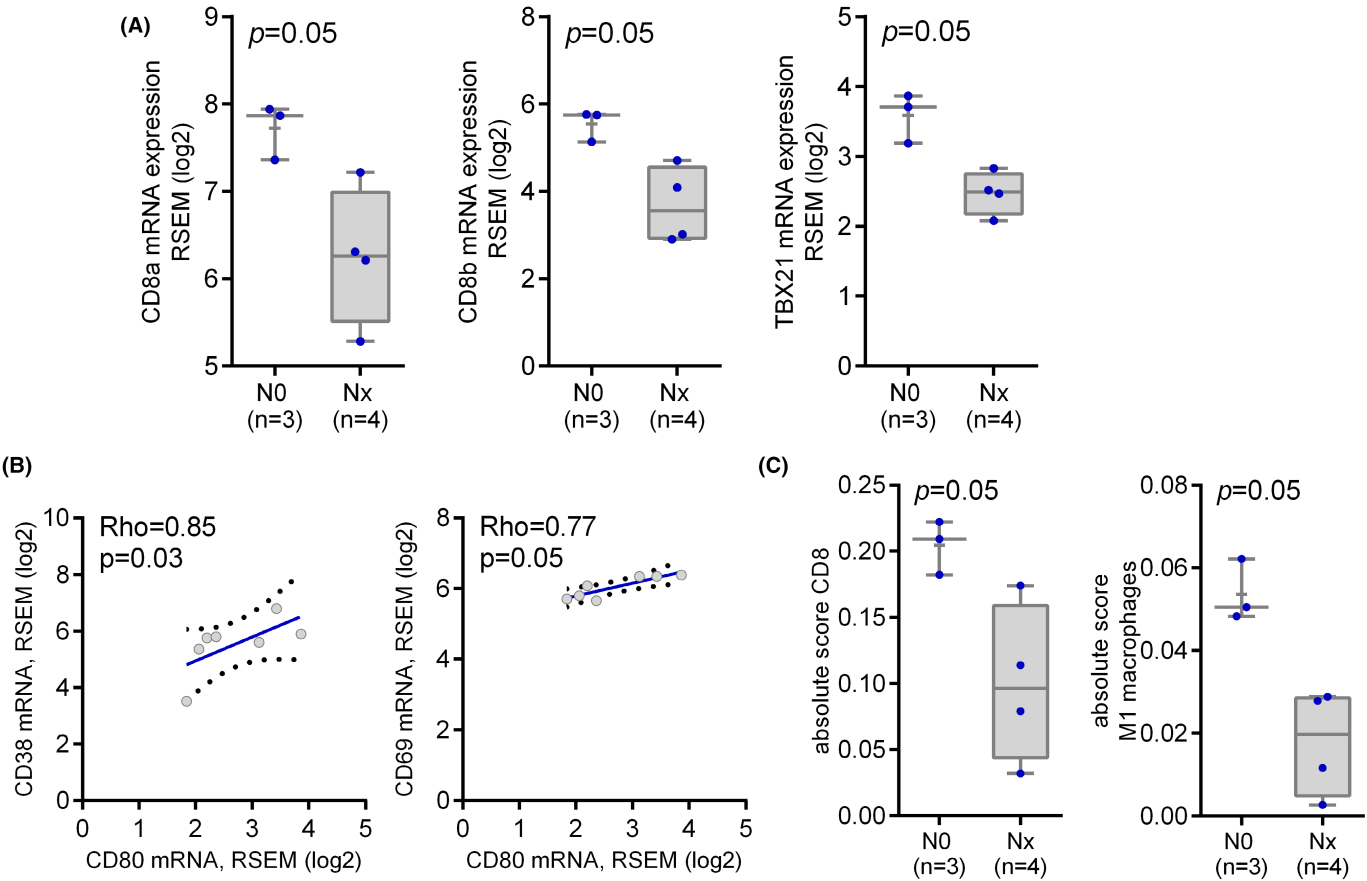
(A) TCGA panCancer Atlas Esophageal Adenocarcinoma‐peritumoral normal tissue: CD8A, CD8B, and TBX21 mRNA expression according to nodal status. (B) Correlation between CD80 and CD38 and CD69 mRNA expression levels in TCGA panCancer Atlas Esophageal Adenocarcinoma‐ peritumoral normal tissue (C) CIBERSORTx Digital Cytometry for CD8 T cells and M2 macrophages populations in TCGA panCancer Atlas Esophageal Adenocarcinoma—peritumoral normal tissue according to nodal status.

Esophageal Adenocarcinoma TCGA PanCancer Atlas dataset comprised 70 therapy‐naive pT1, pT2, or pT3 EAC, with LN involvement identified in 48 patients (68%) (Tables S2 and S3). No significant association between nodal metastasis and the expression of the selected putative immune markers or genomic alterations was found in the intratumoral tissue (data not shown). Among the selected cohort, there were data available also for seven matched adjacent normal tissue samples (four from patients with LN metastasis). Interestingly, in these peritumoral tissue samples *CD8A*, *CD8B*, and *TBX21* expression were reduced in patients with nodal metastasis (*p* = 0.05, *p* = 0.05, and *p* = 0.05, respectively) (Figure 5A). Moreover, *CD80* mRNA levels directly correlated with *CD38* and *CD69* mRNA levels (Rho = 0.85, *p* = 0.03; and Rho = 0.77, *p* = 0.05, respectively), confirming the possible role of CD80 in the pathway leading to T cell activation observed in our series (Figure 5B). Furthermore, immune cell composition was inferred in these peritumoral specimens using CIBERSORTx. Interestingly, the CD8 T cell and M1 macrophage populations resulted significantly enriched in the normal esophageal mucosa of patients without nodal metastasis (*p* = 0.05 and *p* = 0.05, respectively) (Figure 5C).

## 4 DISCUSSION

Preoperative staging in patients undergoing oncological esophagectomy for adenocarcinoma is fundamental for treatment decisions and heavily affects overall survival. However, it has modest accuracy and reliability for pT and pN prediction, and even patients with early EAC (stage I and II) not receiving neoadjuvant therapy have a significant risk of recurrence. A recent study shows a lack of agreement among gastrointestinal pathologists in measuring the depth of submucosal invasion in esophageal endoscopic resections despite formulating a consensus approach for scoring.^34^ Moreover, currently, minimally invasive esophagectomy with two‐field lymphadenectomy is the standard of care for early EAC LN metastasis and sentinel node navigation surgery has been successfully investigated to tailor the extent of lymphadenectomy.^35^ Finally, 15% of patients with cT1b esophageal cancer were found to have a positive nodal disease and, thus, to not be a suitable candidate for endoscopic resection.^36^ Striving to fill the unmet need for reliable predictors of nodal metastasis, we investigated mucosal samples among neoadjuvant therapy‐naïve patients with early EAC to help plan possible adjuvant chemotherapy.

In our series, LN involvement was associated with vascular invasion. Similarly, Barbetta et al. observed that vascular invasion is an independent predicting factor of pathologic nodal involvement.^37^ Moreover, we previously reported that CD31, a marker of angiogenesis, was associated with nodal metastasis in EAC.^38^ In our opinion, histological vascular invasion within primary cancer can be an easily detectable marker of nodal involvement in therapy‐naive EAC. A study assessing the best biopsy pattern to detect it is warranted to validate the use of this marker in clinical practice.

To our knowledge, there is not a known association between most commonly mutated oncogenes and nodal metastases in therapy‐naive EAC. No association was established between mutations in *TP53*, *cMyc*, *p16*, *BRAF*, or MMR proteins and nodal metastasis in our cohort of patients. Similarly, no association between genomic alterations of the most commonly mutated oncogenes and nodal metastasis was observed in the TCGA panCancer Atlas series. Thus, we hypothesized that the main driver of nodal metastasis could be an immune surveillance failure.

In fact, in our series, nodal metastasis was associated with high intratumoral infiltration of lymphomononuclear cells. In our opinion, this is probably due to unbalanced immune surveillance with high infiltration of immunosuppressive cells such as regulatory T cells (Tregs) and M2 macrophages. In fact, in breast cancer, tumor‐draining LN invasion by metastatic cells is associated with local immunosuppression, which can be partially attributed to Treg.^39^ Similarly, the infiltration of Tregs and M2 tumor‐associated macrophages (TAMs) is significantly associated with nodal metastasis and the progression of premalignant lesions to oral squamous cell carcinoma.^40^ Indeed, in our series, nodal metastasis was associated with low levels of activated cytotoxic T cells within the tumor and ROC analysis revealed a good diagnostic accuracy of nodal involvement. Moreover, nodal metastasis tended to be associated with low levels of CD69, MYD88, and TLR4 mRNA expression, T cells, and innate immunity activation markers, respectively, that showed good accuracy in the diagnosis of nodal involvement. Similarly, in early rectal cancers absence of CD8^+^ T‐cell infiltration was strongly associated with nodal metastasis presence.^34^ In a seminal study, Galon and colleageus showed colorectal cancers with signs of early metastatic invasion had lower infiltrates of immune cells and lower transcripts levels of genes related to type 1 helper effector T cells.^35^ In our opinion, our data may suggest that even in esophageal adenocarcinoma the immune surveillance failure within cancer might be associated with the presence of nodal metastasis. Therefore, these findings could provide the basis for larger studies on the clinical use of these biomarkers.

On the other hand, in our series, low levels of activated CD8 T cells in the peritumoral healthy mucosa were associated with nodal metastasis and the ROC curve demonstrated a good diagnostic accuracy of nodal involvement for low levels of activated CD8 T cell infiltration levels. These data seemed to be confirmed also in the normal adjacent to tumor mucosa of esophageal adenocarcinoma TCGA panCancer Atlas, even if their series was extremely limited in terms of sample size. Similarly, in colorectal cancer patients, low numbers of intraepithelial CD8 in the biopsy predicted the presence of nodal metastasis, tumor deposits, and lymphatic and venous invasion in the primary tumor.^36^ Moreover, in peritumoral healthy mucosa, CD80 mRNA levels directly correlated with MYD88, TLR4, and CD69 mRNA levels, suggesting a possible activation pathway. These findings suggest that even in EAC part of the battle for nodal metastasis containment is not only within the tumor but also in the peritumoral healthy tissue.

The main limit of our study is the small sample size that made the differences in the infiltration rate of immune cells and expression levels of immune genes, in somehow, blurred. This study aims at investigating the immune microenvironment in naïve‐therapy EAC and, currently, this is a relatively small subgroup among all EAC patients as it is evident also in the TCGA series. On the other hand, the sample size is adequate for ROC curves analysis, which seems to confirm what is only suggested in comparative analysis.

In conclusion, our study showed that vascular invasion could be a marker of nodal involvement in therapy‐naive EAC that can be easily detected with standard histology. Our data suggest that immune surveillance failure might be the main driver of nodal metastasis onset, whereas no association between mutations in common oncogenes and nodal metastasis was observed. Finally, our findings suggest that nodal metastasis containment also seems to involve the immune microenvironment of the peritumoral healthy tissue. These findings could provide the basis for larger studies on the clinical use of these biomarkers.

## AUTHOR CONTRIBUTIONS

**Andromachi Kotsafti:** Conceptualization (equal); investigation (equal); writing – original draft (equal); writing – review and editing (equal). **Matteo Fassan:** Conceptualization (equal); investigation (equal); writing – original draft (equal); writing – review and editing (equal). **Francesco Cavallin:** Data curation (equal); writing – review and editing (equal). **Valentina Angerilli:** Investigation (equal). **Luca Saadeh:** Investigation (equal). **Matteo Cagol:** Investigation (equal). **Rita Alfieri:** Investigation (equal). **Pierluigi Pilati:** Funding acquisition (equal); investigation (equal); supervision (equal). **Carlo Castoro:** Funding acquisition (equal); investigation (equal); supervision (equal). **Ignazio Castagliuolo:** Funding acquisition (equal); investigation (equal); supervision (equal). **Melania Scarpa:** Conceptualization (equal); investigation (equal); visualization (equal); writing – review and editing (equal). **Marco Scarpa:** Conceptualization (equal); data curation (equal); funding acquisition (equal); project administration (equal); supervision (equal); writing – original draft (equal); writing – review and editing (equal).

## FUNDING INFORMATION

Open access funding provided by BIBLIOSAN.

## CONFLICT OF INTEREST

The authors have no conflict of interest.

## Supporting information


Figure S1

Table S1
Table S2
Table S3
Table S4

## Data Availability

The data that support the findings of this study are available from the corresponding author upon reasonable request.
