# Supplementary figures and images for: Tumor immune microenvironment in therapy‐naive esophageal adenocarcinoma could predict the nodal status
Source: Cancer Med. 2022 Oct 25;12(5):5526–35. doi: 10.1002/cam4.5386 (PMC10028023; doi:10.1002/cam4.5386)

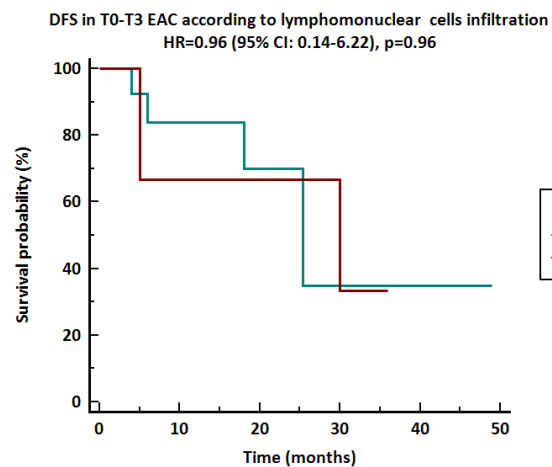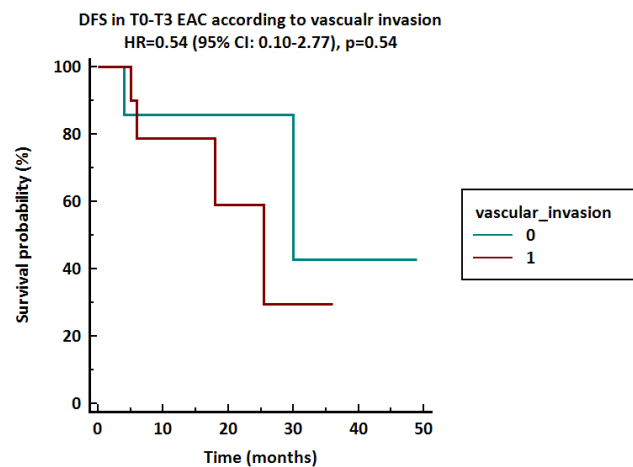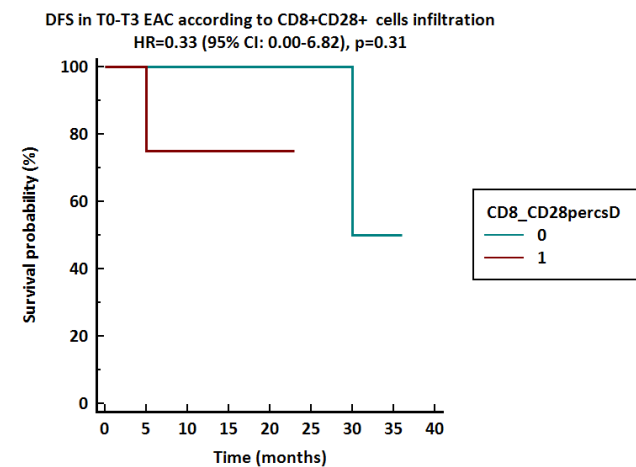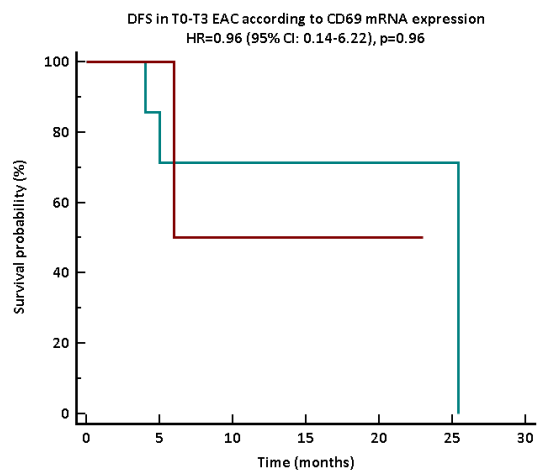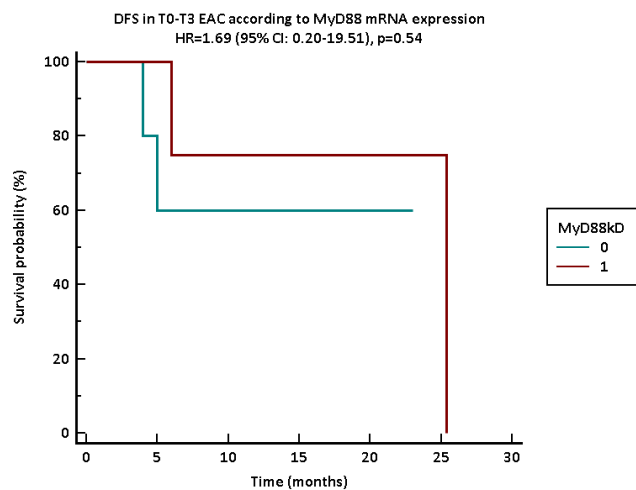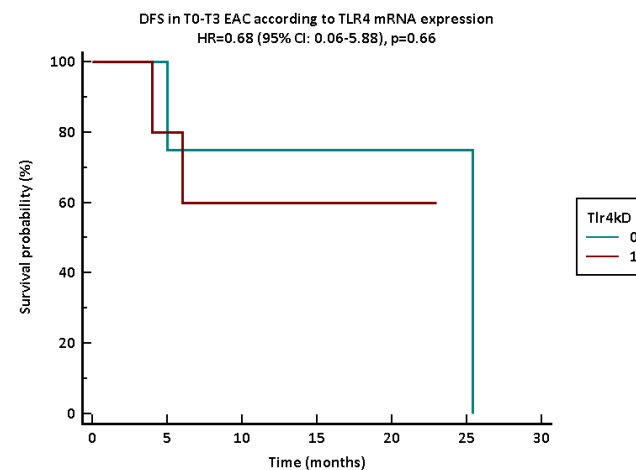

**Supplementary Figure 1**

Supplement: Supplementary file 1 — Figure S1 [file CAM4-12-5526-s002.pdf]
