# Supplementary material for: Tumor immune microenvironment in therapy‐naive esophageal adenocarcinoma could predict the nodal status
Source: Cancer Med. 2022 Oct 25;12(5):5526–35. doi: 10.1002/cam4.5386 (PMC10028023; doi:10.1002/cam4.5386)
Supplement: Supplementary file 2 — Table S1 Table S2 Table S3 Table S4 [file CAM4-12-5526-s001.docx]

**SUPPLEMENTARY TABLES**

**Supplementary Table S1.** Characteristics of the study population and comparison of the demographic data between the group of patients who had neoadjuvant therapy and accepted to participate to MICCE1 project and the selected group (total n=151)

| **Characteristics** | **Neoadjuvant therapy** | | ***p* values** |
| --- | --- | --- | --- |
|  | No (study population)  (n=30) | Yes  (n=121) |  |
| *Age*, median (IQR) | 72 (IQR 58.5-78.5) | 62 (IQR 54-67.2) | 0.014 |
| *Gender*  Female  Male | 4  26 | 12  109 | 0.740 |
| *Associated to Barrett epithelium*  Yes  No | 9  21 | 18  103 | 0.064 |
| *Cancer stage*  Tis/T0  T1  T2  T3 | 2  11  6  11 | 29  5  33  52 | <0.001 |
| *Cancer grading*  *G1*  *G2*  *G3* | 5  15  10 | 22  47  52 | 0.522 |
| *Nodal metastasis*  Median number of resected lymph nodes  Median number of nodal metastasis | 28 (IQR 18-33.7)  0 (IQR 0-1) | 25 (IQR 20-33)  0 (IQR 0-3) | 0.908  0.412 |
| *Surgical radicality*  R0  R1 | 30  0 | 121  0 | 0.999 |

**Supplementary Table S2.** Characteristics of the TCGA study population (n=70).

| **Characteristics** | **Lymph nodes**  **involvement** | | ***p* values** |
| --- | --- | --- | --- |
|  | Absent  (n=22)  N0=22 | Present  (n=48)  N1=37  N2=6  N3=5 |  |
| *Age*, median (IQR) | 72.5  (60-77) | 68.5  (59-77) | 0.372 |
| *Gender*  Female  Male | 4  18 | 6  42 | 0.535 |
| *Cancer stage*  T1  T2  T3 | 14  1  7 | 9  9  30 | 0.001 |
| *Metastasis stage*  M0  M1  MX  NA | 17  2  3  0 | 33  3  8  4 | 0.735 |

**Supplementary Table S3.** Characteristics of the TCGA study population with matched normal tissue adjacent to tumor (n=7).

| **Characteristics** | **Lymph nodes**  **involvement** | | ***p* values** |
| --- | --- | --- | --- |
|  | Absent  (n=3)  N0=3 | Present  (n=4)  N1=2  N2=1  N3=1 |  |
| *Age*, median (IQR) | 79  (74.5-81) | 69  (62-75) | 0.171 |
| *Gender*  Female  Male | 2  1 | 0  4 | 0.062 |
| *Cancer stage*  T1  T2  T3 | 3  0  0 | 2  1  1 | 0.242 |
| *Metastasis stage*  M0  M1  MX  NA | 2  0  1  0 | 2  0  1  1 | 0.999 |

**Supplementary Table S4**. Sensitivity and specificity analysis.

| **Variable** | **Area under the ROC curve** | **95% CI** | **Criterion** | **Sensitivity** | **95% CI** | **Specificity** | **95% CI** |
| --- | --- | --- | --- | --- | --- | --- | --- |
| CD38 mRNA levels in cancer | 0,74 | 0,456 to 0,924 | <=0,0065 * | 100 | 48,0 - 100,0 | 60 | 26,4 - 87,6 |
| CD69 mRNA levels in cancer | 0,76 | 0,476 to 0,935 | <=0,0102 * | 100 | 48,0 - 100,0 | 50 | 18,9 - 81,1 |
| CD8+CD28+ cell rate in cancer | 0,817 | 0,481 to 0,971 | <=2,4 * | 100 | 48,0 - 100,0 | 66,67 | 22,7 - 94,7 |
| CD8+CD28+ cell rate in healthy mucosa | 0,8 | 0,463 to 0,966 | <=1,9 * | 100 | 48,0 - 100,0 | 66,67 | 22,7 - 94,7 |
| MyD88 mRNA levels in cancer | 0,8 | 0,519 to 0,954 | <=0,0385 * | 100 | 48,0 - 100,0 | 60 | 26,4 - 87,6 |
| Tlr4 mRNA levels in cancer | 0,8 | 0,519 to 0,954 | <=0,0001 * | 100 | 48,0 - 100,0 | 60 | 26,4 - 87,6 |
